# Clinical and Microbiological Profile of Infections in Hospitalized Patients with Sickle Cell Disease: A Tertiary Care Centre Experience from Central India

**DOI:** 10.7759/cureus.94286

**Published:** 2025-10-10

**Authors:** Ananta Mishra, Pankaj K Kannauje, Vinay Pandit

**Affiliations:** 1 General Medicine, All India Institute of Medical Sciences, Raipur, Raipur, IND

**Keywords:** antimicrobial resistance, infections, procalcitonin, risk score, sickle cell disease, tribal population

## Abstract

Background

Sickle cell disease (SCD) is highly prevalent among India's tribal populations, with infections being a major cause of morbidity and mortality. Current data on causative pathogens, antimicrobial resistance patterns, and clinical predictors of outcomes in Indian SCD patients remain limited. This study aims to characterize the clinical and microbiological profile of infections in hospitalized SCD patients and identify predictors of adverse outcomes.

Methods

An ambispective observational study was conducted at a tertiary care center in central India from December 2018 to December 2021 (retrospective phase, n=61) and January 2022 to June 2023 (prospective phase, n=150), totaling 211 SCD patients with confirmed infections. Data were analyzed using IBM SPSS Statistics software, version 25.0 (IBM Corp., Armonk, NY). An exploratory secondary analysis developed a preliminary risk score for predicting adverse outcomes using multivariate logistic regression and receiver operating characteristic (ROC) curve analysis.

Results

Among 211 hospitalized SCD patients (median age 21 years, range two to 65 years), respiratory infections were most common (34.6%), followed by acute undifferentiated febrile illness (19.9%), bone and joint infections (9.5%), and bloodstream infections (9%). Among 32 gram-negative isolates, 50% were extended-spectrum β-lactamase (ESBL) producers with 100% susceptibility to polymyxins. Fever was present in 86.7% of patients. Procalcitonin levels >2 ng/mL were significantly associated with adverse outcomes (p=0.04). Overall mortality was 6.6% (14 deaths), with respiratory infections accounting for the highest number of deaths (8/73, 11.0%). An exploratory SCD Infection Risk Score (SCD-IRS) incorporating age, hemoglobin level, total leucocyte count, and procalcitonin demonstrated moderate discriminative ability with an area under the curve (AUC) of 0.78 (95% CI: 0.65-0.91, p<0.001) but lacks internal validation due to sample size constraints.

Conclusions

Respiratory infections predominate among hospitalized SCD patients in central India. High antimicrobial resistance poses significant treatment challenges. Elevated procalcitonin levels (>2 ng/mL) are significantly associated with adverse outcomes. The preliminary SCD-IRS shows potential for risk stratification but is strictly exploratory and requires validation in larger, multi-institutional studies before any clinical application. Strengthening vaccination coverage, implementing antimicrobial stewardship programs, and developing validated risk assessment tools are essential to reduce infection-related morbidity and mortality in this vulnerable population.

## Introduction

Sickle cell disease (SCD), characterized by a genetic mutation in the beta-globin gene leading to abnormal hemoglobin S production, results in red blood cell sickling, hemolytic anemia, vaso-occlusion, and multi-organ complications [1]. Globally, approximately 300,000-400,000 infants are born annually with severe hemoglobin disorders [1].

In India, SCD is particularly prevalent among tribal populations concentrated in the "sickle cell belt" spanning central and western states, including Madhya Pradesh, Maharashtra, Odisha, Gujarat, Rajasthan, Jharkhand, Chhattisgarh, Andhra Pradesh, West Bengal, and Karnataka [2]. The prevalence of sickle cell trait varies significantly across states, ranging from 10% to 33% in various tribal groups [3-7].

SCD patients exhibit heightened susceptibility to infections due to functional asplenia, impaired immune responses, and chronic hemolysis [8]. Infections remain the leading causes of hospitalization and mortality, particularly in children and immunocompromised adults. Despite this clinical significance, comprehensive data on microbiological spectrum, antimicrobial resistance patterns, and validated risk assessment tools among Indian SCD patients, especially tribal populations, remain scarce. Current risk assessment in SCD patients relies primarily on clinical judgment and individual laboratory parameters, lacking standardized, validated tools for predicting adverse outcomes. The integration of multiple clinical and laboratory variables into a practical scoring system could significantly improve clinical decision-making and resource allocation.

This study aims to characterize the clinical, epidemiological, and microbiological profiles of infections in hospitalized SCD patients at a tertiary care center in Chhattisgarh, and as an exploratory secondary objective, identify potential predictors of adverse outcomes that could guide future risk assessment tool development in this vulnerable population.

## Materials and methods

Study design and setting

An ambispective observational study was conducted in the general medicine and pediatrics departments at All India Institute of Medical Sciences (AIIMS), Raipur, a tertiary care center in Chhattisgarh, India, involving both ward and ICU admissions. The institution serves as a regional referral center for tribal populations with high SCD prevalence, providing specialized care for patients from across central India. The study included a retrospective phase from December 2018 to December 2021 (36 months, n=61 patients) and a prospective phase from January 2022 to June 2023 (18 months, n=150 patients), with a total final sample of 211 patients. The data lock date for final analysis was July 2023. Sample size was calculated using the following formula

$n = \frac{Z^{2}P(1 - P)}{d^{2}}$

where Z = 1.96 (95% confidence), P = 0.10 (assumed prevalence), and d = relative precision.

In the retrospective phase, 69 eligible patients were identified; eight were excluded due to incomplete records, and 61 were included. In the prospective phase, 162 eligible patients were approached; 12 declined consent, and 150 were included. All consecutive hospitalized SCD patients meeting eligibility criteria during these periods were screened for inclusion.

Inclusion criteria

All SCD patients admitted with clinical or laboratory evidence of infection at admission or during hospital stay, age ≥1 year (minimum age: two years; maximum age: 65 years), and confirmed SCD diagnosis by hemoglobin electrophoresis using cellulose acetate membrane at alkaline pH or high-performance liquid chromatography (HPLC) showing HbSS pattern were included.

Exclusion criteria

Patients refusing consent, those with other immunocompromised conditions (HIV, malignancy, uncontrolled diabetes, dialysis for non-sickle nephropathy), children <1 year of age, and those with incomplete medical records were excluded.

Admission criteria

Patients were admitted to the hospital based on one or more of the following criteria: (1) CURB-65 score ≥2 for respiratory infections (confusion, Urea >7 mmol/L, respiratory rate ≥30 breaths/min, blood pressure <90/60 mmHg, age ≥65 years), (2) quick Sequential Organ Failure Assessment (qSOFA) score ≥2 for suspected sepsis (respiratory rate ≥22 breaths/min, altered mentation, systolic blood pressure ≤100 mmHg), (3) severe anemia (hemoglobin <7.0 g/dL) with fever, (4) vaso-occlusive crisis with concurrent infection requiring parenteral analgesics, (5) clinical instability requiring monitored care or ICU admission, or (6) specific infection requiring hospitalization (osteomyelitis, pyelonephritis, severe pneumonia).

Infection definition

Evidence of infection was defined as one or more of the following: (1) temperature >38°C or <36°C with identifiable clinical focus of infection, (2) positive microbiological culture from sterile site (blood, CSF, pleural fluid) or non-sterile site with clinical correlation, (3) radiological evidence of infection (pneumonic consolidation on chest X-ray, abscess on ultrasound/CT), (4) positive rapid diagnostic test for tropical fevers (malaria, dengue, scrub typhus), or (5) elevated inflammatory markers (CRP >50 mg/L or procalcitonin >0.5 ng/mL) with compatible clinical syndrome.

Data collection

Structured questionnaires recorded sociodemographic data, including age, sex, residential distance, marital and family history, socioeconomic status, vaccination status, hospital admission frequency, and medication use (antibiotics, hydroxyurea). Clinical history and examination findings were systematically documented.

Laboratory investigations

Comprehensive laboratory workup was performed for all patients including hematology (complete blood count with peripheral blood smear examination to assess hemoglobin levels, leucocyte and platelet counts, and red blood cell morphology), infectious disease markers (rapid diagnostic tests and microscopy for malaria, dengue NS1 antigen and IgM antibodies, scrub typhus IgM antibodies), microbiological studies (cultures from blood, urine, sputum, and pus samples, followed by antimicrobial susceptibility testing), radiology (chest X-rays for all patients, abdominal ultrasound when indicated, CT or MRI scans for specific clinical scenarios), cerebrospinal fluid analysis in patients with suspected CNS infections (cell count, protein, glucose, microbiological examination), fungal investigations (biomarkers and tissue biopsy when clinically appropriate), and procalcitonin.

Microbiological methods

Culture and antimicrobial susceptibility testing were performed using automated blood culture system (BacT/ALERT 3D, bioMérieux, Durham, NC, USA) with standard aerobic and anaerobic bottles for blood cultures, standard MacConkey agar and blood agar incubated at 37°C for 24-48 hours for other cultures, conventional biochemical tests, Kirby-Bauer disk diffusion method per Clinical and Laboratory Standards Institute (CLSI) guidelines for antimicrobial susceptibility testing, combination disk method per CLSI criteria for ESBL detection, and American Type Culture Collection (ATCC) reference strains for quality control validation.

Study variables

Primary outcome variables included in-hospital mortality, ICU admission requirement, length of hospital stay exceeding 14 days, and a composite adverse outcome defined as death, ICU admission, OR prolonged hospitalization (>14 days). Secondary variables included prior antibiotic use (within one week of admission), vaccination status (Universal Immunization Program vaccines and special vaccines: pneumococcal and Haemophilus influenzae type b), and medication history (folic acid and hydroxyurea).

Data handling and missing values

Complete-case analysis was used for the risk score development. Patients with missing procalcitonin values (n=18, 8.5%) were excluded from multivariate modeling. All other baseline and outcome variables had <5% missing data. No imputation methods were used.

Development of preliminary risk assessment

Following the primary analysis, an exploratory secondary analysis was conducted to identify potential predictors of adverse outcomes that could inform future risk assessment tool development. All variables with p<0.10 in univariate analysis were considered for inclusion as candidate variables: age >30 years, hemoglobin <7 g/dL, total leucocyte count >15,000/μL, procalcitonin >2 ng/mL, presence of bloodstream infection, prior antibiotic use (within one week), and distance from hospital >100 km. The model-building strategy involved multivariate logistic regression with backward elimination (retention threshold p<0.05). The events-per-variable (EPV) ratio was 36 adverse outcomes ÷ 5 final predictors = 7.2 EPV. Point assignment was based on β-coefficients rounded to the nearest integer. Critical limitation was that no internal validation (bootstrap resampling or split-sample) was performed due to limited sample size.

The model specification for reproducibility wass Logit(adverse outcome) = -3.45 + 1.34(Age>30) + 1.43(Hb<7) + 1.13(TLC>15,000) + 1.72(PCT>2) + 1.07(Bloodstream infection). Point assignment was as follows: age >30 years: 3 points (β=1.34), hemoglobin <7 g/dL: 3 points (β=1.43), total leucocyte count >15,000/μL: 2 points (β=1.13), procalcitonin >2 ng/mL: 4 points (β=1.72), bloodstream infection present: 2 points (β=1.07), with a total possible score range of 0-14 points. Model discrimination was assessed using receiver operating characteristic (ROC) curve analysis. Calibration was assessed using the Hosmer-Lemeshow goodness-of-fit test.

Statistical analysis

Data were entered in Microsoft Excel 2016 (Microsoft Corporation, Redmond, WA, USA) and analyzed using IBM SPSS Statistics software version 25.0 (IBM Corp., Armonk, NY, USA). Descriptive statistics included means, medians, and proportions for baseline characteristics. Categorical variables were compared using chi-square or Fisher's exact test for expected cell counts < 5. The Mann-Whitney U test was used for non-normally distributed continuous variables. Statistical significance was set at p<0.05.

## Results

Demographic characteristics

A total of 211 SCD patients were analyzed, with a median age of 21 years (interquartile range 15-29 years; range two to 65 years). Male patients constituted 116 (55%) of the study population, while female patients accounted for 95 (45%). The age distribution revealed that half of the patients (106, 50.2%) were young adults between 15 and 30 years, with pediatric patients under 15 years comprising 78 (37%) of the cohort, and only 27 (12.8%) being older than 30 years. The majority of patients (187, 88.4%) belonged to tribal communities, reflecting the high prevalence of SCD in these populations. Most patients resided within 50 kilometers of the hospital (96, 45.5%), though a significant proportion (61, 28.9%) travelled more than 100 kilometers for care, highlighting the limited access to specialized healthcare in these regions (Table 1).

**Table 1:** Demographic and Clinical Characteristics of the Study Population (n=211) *Percentage calculated from married patients (n=73) km: kilometers; SCD: sickle cell disease; UIP: Universal Immunization Programme; Hib: *Haemophilus influenzae type b*

Characteristic	n (%)
Age groups	
<15 years	78 (37.0)
15-30 years	106 (50.2)
>30 years	27 (12.8)
Gender	
Male	116 (55.0)
Female	95 (45.0)
Distance from the hospital	
≤50 km	96 (45.5)
50-100 km	54 (25.6)
>100 km	61 (28.9)
Blood group	
O+	90 (42.7)
A+	58 (27.5)
B+	42 (19.9)
AB+	21 (10.0)
Socioeconomic status	
Upper-lower	106 (50.2)
Upper-middle	66 (31.3)
Lower-middle	24 (11.4)
Upper	15 (7.1)
Marital status	
Married	73 (34.6)
Spouse with SCD	13 (17.8)*
Unmarried	138 (65.4)
Family history	
Siblings with SCD	183 (87.0)
Consanguineous marriage	8 (3.8)
Vaccination status	
UIP vaccines	188 (89.1)
Special vaccines (Hib/Pneumococcal)	55 (26.1)
Prior antibiotic use (1 week)	88 (41.7)

Spectrum of infections

Classification Methodology

Infections were classified by primary etiological agent (bacterial/viral/fungal/mycobacterial) and anatomical site. Radiological findings and microbiological confirmation were used where available. For acute undifferentiated febrile illness, no specific focus could be identified despite systematic evaluation (Table 2).

**Table 2:** Spectrum of Infections in Hospitalized SCD Patients (n=211) *All bloodstream infections were community-acquired; no catheter-related bloodstream infections (CRBSI) or central line-associated bloodstream infections (CLABSI) were documented in this cohort. SCD: sickle cell disease

Type of Infection	n (%)	Etiological Agent
1. Respiratory infections	73 (34.6)	Mixed bacterial/viral/mycobacterial
Bacterial pneumonia	52 (71.2)	Bacterial
Pneumonia with pleural effusion	9 (12.3)	Bacterial
COVID-19 pneumonia	4 (5.5)	Viral
Pulmonary tuberculosis	4 (5.5)	Mycobacterial
TB with pleural effusion	2 (2.7)	Mycobacterial
Others	2 (2.7)	Mixed
2. Acute undifferentiated febrile illness	42 (19.9)	Unknown/presumed bacterial
3. Bone and joint infections	20 (9.5)	Bacterial
Osteomyelitis	15 (75.0)	Bacterial
Septic arthritis	5 (25.0)	Bacterial
4. Septicemia/Bloodstream infections*	19 (9.0)	Bacterial (culture-confirmed)
5. Hepatobiliary infections	14 (6.6)	Viral/bacterial
Hepatitis B	6 (42.9)	Viral
Hepatitis A	5 (35.7)	Viral
Splenic abscess	3 (21.4)	Bacterial
6. Gastrointestinal infections	4 (1.9)	Mixed bacterial/parasitic
Acute gastroenteritis	4 (100.0)	
7. Urinary tract infections	18 (8.5)	Bacterial
Cystitis	13 (72.2)	Bacterial
Pyelonephritis	5 (27.8)	Bacterial
8. Tropical fevers	12 (5.7)	Parasitic/bacterial
Malaria	7 (58.3)	Parasitic
Scrub typhus	3 (25.0)	Bacterial
Dengue	2 (16.7)	Viral
9. Others	9 (4.3)	Mixed bacterial/viral
Leprosy (Hansen's disease-type 1 reversal reaction)	4 (44.4)	Bacterial
Mumps	1 (11.1)	Viral
Others	4 (44.4)	Mixed

Clinical manifestations and laboratory parameters

Fever was the most common presenting symptom, occurring in 183 patients (86.7%), which is expected given the infectious nature of the conditions studied. Pallor was nearly universal, affecting 209 patients (99.0%), reflecting the chronic anemia characteristic of SCD. Respiratory symptoms were prominent, with cough present in 55 patients (26.1%), breathlessness in 42 patients (19.9%), and chest pain in 38 patients (18.0%). Abdominal pain was reported by 35 patients (16.6%), often related to vaso-occlusive episodes or splenic complications.

Physical examination findings revealed icterus in 95 patients (45.0%), indicating ongoing hemolysis or hepatic involvement. Hepatomegaly was documented in 84 patients (39.8%), while splenomegaly was present in 70 patients (33.2%). Lymphadenopathy was observed in 28 patients (13.3%), suggesting a systemic inflammatory response or specific infections. Functional or surgical splenectomy was present in five patients (2.4%), with autosplenectomy being more common than surgical removal (three vs. two cases, respectively).

Laboratory parameters revealed significant anemia, with most patients having hemoglobin levels between 7.0 and 9.9 g/dL in 145 (68.7%), while 45 (21.3%) had severe anemia with hemoglobin <7.0 g/dL, and only 21 (10%) had relatively preserved hemoglobin ≥10.0 g/dL. Leukocytosis was common, with 136 (64.5%) of patients having total leukocyte counts >11,000/μL, reflecting both the inflammatory response to infection and the baseline elevated white cell count in SCD patients. Leukopenia (<4,000/μL) was present in 19 (9%) of patients, indicating possible bone marrow suppression or overwhelming infection.

Procalcitonin levels provided important prognostic information, with 78 (37%) of patients having levels <0.5 ng/mL, 89 (42.2%) having intermediate levels (0.5-2.0 ng/mL), and 44 (20.9%) having markedly elevated levels >2.0 ng/mL, the latter group showing significantly worse outcomes (Table 3).

**Table 3:** Clinical Manifestations and Laboratory Parameters (n=211) *Percentage calculated from patients with splenectomy (n=5). Note: Clinical manifestations represent presentation at admission. Pallor was nearly universal (99%), reflecting the chronic hemolytic anemia characteristic of SCD rather than acute infection-related findings. SCD: sickle cell disease

Parameter	n (%)
Clinical symptoms	
Fever	183 (86.7)
Cough	55 (26.1)
Breathlessness	42 (19.9)
Chest pain	38 (18.0)
Abdominal pain	35 (16.6)
Physical signs	
Pallor	209 (99.0)
Icterus	95 (45.0)
Hepatomegaly	84 (39.8)
Splenomegaly	70 (33.2)
Lymphadenopathy	28 (13.3)
Splenectomy	5 (2.4)
- Surgical	2 (40.0)*
- Autosplenectomy	3 (60.0)*
Hemoglobin levels (g/dL)	
<7.0	45 (21.3)
7.0-9.9	145 (68.7)
≥10.0	21 (10.0)
Total leucocyte count	
<4,000	19 (9.0)
4,000-11,000	56 (26.5)
>11,000	136 (64.5)
Procalcitonin levels	
<0.5 ng/mL	78 (37.0)
0.5-2.0 ng/mL	89 (42.2)
>2.0 ng/mL	44 (20.9)

Antimicrobial resistance

Tables 4, 5, present organism-specific antimicrobial susceptibility patterns for 32 gram-negative isolates. The antimicrobial susceptibility testing revealed concerning resistance patterns that have significant clinical implications. Colistin (polymyxin E) demonstrated universal susceptibility, with all 32 isolates (100%) showing sensitivity across all organisms, *Escherichia coli* (*E. coli*) (n=12), and *Klebsiella *spp. (n=11), *Pseudomonas *spp. (n=8), *Acinetobacter *spp. (n=7), and *Salmonella *spp. (n=6), making it a last-resort therapeutic option for multidrug-resistant infections. However, colistin is nephrotoxic (polymyxin B has less renal toxicity but was not differentiated in all susceptibility reports), which is particularly concerning in SCD patients who may have baseline renal complications from repeated vaso-occlusive episodes. Among *E. coli* isolates, ampicillin showed 75% susceptibility, gentamicin 66.7%, ciprofloxacin 50%, and ceftriaxone only 25%, with ESBL production detected in six isolates (50%) and multidrug resistance in eight isolates (66.7%). *Klebsiella *species demonstrated even higher resistance rates, with amikacin showing 81.8% susceptibility, ciprofloxacin 45.5%, and cefotaxime only 18.2%, with ESBL production in seven isolates (63.6%) and multidrug resistance in nine isolates (81.8%). Pseudomonas species showed piperacillin-tazobactam susceptibility of 75%, amikacin 62.5%, and ciprofloxacin 37.5%, with ESBL production in three isolates (37.5%) and multidrug resistance in seven isolates (87.5%). *Acinetobacter *species demonstrated the worst resistance profile, with amikacin showing only 42.9% susceptibility and most other agents showing <30% susceptibility, with ESBL production in four isolates (57.1%) and universal multidrug resistance (100%). *Salmonella *species showed relatively better susceptibility patterns with ceftriaxone (66.7%) and ciprofloxacin (83.3%), with ESBL production in two isolates (33.3%) and multidrug resistance in three isolates (50%) (Table 4). Appendix A provides a detailed distribution of the infection sites.

**Table 4:** Organism-Specific Antimicrobial Susceptibility Patterns ESBL: extended-spectrum β-lactamase; MDR: multi-drug resistant (resistant to ≥3 antibiotic classes); gram-negative isolates (n=32)

Organism	n	Antimicrobial Agent	Susceptible n (%)	Resistant n (%)	ESBL+ n (%)	MDR n (%)
Escherichia coli	12	Polymyxin B/Colistin	12 (100.0)	0 (0.0)	6 (50.0)	8 (66.7)
		Ampicillin	9 (75.0)	3 (25.0)		
		Gentamicin	8 (66.7)	4 (33.3)		
		Ciprofloxacin	6 (50.0)	6 (50.0)		
		Ceftriaxone	3 (25.0)	9 (75.0)		
*Klebsiella *spp.	11	Polymyxin B/Colistin	11 (100.0)	0 (0.0)	7 (63.6)	9 (81.8)
		Amikacin	9 (81.8)	2 (18.2)		
		Ciprofloxacin	5 (45.5)	6 (54.5)		
		Cefotaxime	2 (18.2)	9 (81.8)		
*Pseudomonas *spp.	8	Polymyxin B/Colistin	8 (100.0)	0 (0.0)	3 (37.5)	7 (87.5)
		Piperacillin-tazobactam	6 (75.0)	2 (25.0)		
		Amikacin	5 (62.5)	3 (37.5)		
		Ciprofloxacin	3 (37.5)	5 (62.5)		
		Ceftazidime	2 (25.0)	6 (75.0)		
*Acinetobacter *spp.	7	Polymyxin B/Colistin	7 (100.0)	0 (0.0)	4 (57.1)	7 (100.0)
		Amikacin	3 (42.9)	4 (57.1)		
		All others	<30% susceptibility			
*Salmonella *spp.	6	Polymyxin B/Colistin	6 (100.0)	0 (0.0)	2 (33.3)	3 (50.0)
		Ceftriaxone	4 (66.7)	2 (33.3)		
		Ciprofloxacin	5 (83.3)	1 (16.7)		

**Table 5:** Antimicrobial Susceptibility Pattern of Gram-negative Isolates (n=32) *Colistin (polymyxin E) is the nephrotoxic polymyxin; polymyxin B has less renal toxicity but was not differentiated in all susceptibility reports; **Macrolide susceptibility testing was performed to assess coverage for atypical pathogens in mixed respiratory infections. Extended-spectrum β-lactamase (ESBL) production: 16/32 (50.0%)

Antibiotic Class	Specific Antibiotics	Susceptible n (%)	Resistant n (%)
Polymyxins	Colistin*, polymyxin B	32 (100.0)	0 (0.0)
Beta-lactams	Ampicillin, penicillin	26 (81.3)	6 (18.8)
Macrolides**	Azithromycin, erythromycin	25 (78.1)	7 (21.9)
Aminoglycosides	Gentamicin, amikacin	24 (75.0)	8 (25.0)
Beta-lactamase inhibitors	Amoxicillin-clavulanate	21 (65.6)	11 (34.4)
Quinolones	Ciprofloxacin, levofloxacin	16 (50.0)	16 (50.0)
Cephems	Ceftriaxone, cefotaxime	6 (18.8)	26 (81.3)
Folate inhibitors	Cotrimoxazole	6 (18.8)	26 (81.3)

The organism-specific resistance patterns reveal important therapeutic considerations. *E. coli *and *Klebsiella *species demonstrated high rates of ESBL production (50% and 63.6%, respectively) and multidrug resistance (66.7% and 81.8%), with particularly concerning resistance to third-generation cephalosporins (75%-81.8%). *Pseudomonas *and *Acinetobacter *species showed near-universal multidrug resistance (87.5% and 100%), limiting therapeutic options to colistin and carbapenems. The relatively lower resistance rates in *Salmonella *species (50% multidrug resistant, 33.3% ESBL) may reflect less selective pressure in community-acquired enteric infections. These patterns necessitate de-escalation strategies based on cultural results rather than prolonged empirical broad-spectrum therapy (Table 5).

Clinical outcomes and mortality analysis

The overall clinical outcomes demonstrated that the majority of patients (175, 82.9%) were discharged without significant disability, indicating successful management of their infections. However, 15 patients (7.1%) were discharged with some form of disability, likely related to complications from severe infections or underlying SCD complications. The overall mortality rate was 14 (6.6%) patients, which reflects the serious nature of infections in this immunocompromised population. Seven patients (3.3%) left against medical advice, which is a common challenge in managing patients from remote tribal areas who may face financial or social pressures to return home.

When analyzing mortality by infection type, respiratory infections accounted for the highest absolute number of deaths (11.0% case fatality rate), with eight fatalities among 73 patients. Bloodstream infections showed the highest case fatality rate at 15.8% (three deaths among 19 patients), reflecting the severity of systemic infections. Bone and joint infections, gastrointestinal infections, and urinary tract infections each contributed one death, representing case fatality rates of 5.0%, 5.6%, and 5.6%, respectively.

The prognostic significance of procalcitonin was clearly demonstrated in the mortality analysis. Among the 14 patients who died, 9 (64.3%) had procalcitonin levels >2 ng/mL, while among the 197 survivors, only 35 (17.8%) had similarly elevated procalcitonin levels. This difference was statistically significant (p=0.04), establishing procalcitonin as a valuable biomarker for risk stratification in SCD patients with infections.

Hospital length of stay analysis revealed that 89 patients (42.2%) had relatively short stays of less than seven days, while 78 patients (37.0%) required seven to 14 days of hospitalization. A significant proportion (44 patients, 20.9%) required prolonged hospitalization exceeding 14 days, indicating severe infections or complications. The mean hospital stay was 9.2 ± 6.8 days, which is considerably longer than typical infection-related hospitalizations, reflecting the complex nature of managing infections in SCD patients (Table 6).

**Table 6:** Clinical Outcomes and Mortality Analysis (n=211) *Statistically significant (p<0.05)

Outcome	n (%)
Overall outcomes	
Discharged without disability	175 (82.9)
Discharged with disability	15 (7.1)
Death	14 (6.6)
Left against medical advice (LAMA)	7 (3.3)
Mortality by infection type	
Respiratory infections	8/73 (11.0)
Bloodstream infections	3/19 (15.8)
Bone and joint infections	1/20 (5.0)
Gastrointestinal infections	1/18 (5.6)
Urinary tract infections	1/18 (5.6)
Procalcitonin and mortality	
Procalcitonin >2 ng/mL with death	9/14 (64.3)
Procalcitonin >2 ng/mL survivors	35/197 (17.8)
p-value	0.04*
Hospital stay duration	
<7 days	89 (42.2)
7-14 days	78 (37.0)
>14 days	44 (20.9)
Mean ± SD (days)	9.2 ± 6.8

Exploratory development of SCD infection risk assessment

Univariate Analysis for Risk Factors

The univariate analysis comparing patients with and without adverse outcomes (composite of death, ICU admission, or hospital stay >14 days) revealed several significant associations that informed the subsequent multivariate modeling. Age greater than 30 years emerged as a strong predictor, being present in 12 of 36 patients (33.3%) with adverse outcomes compared to only 15 of 175 patients (8.6%) without adverse outcomes (p<0.001). This finding likely reflects the cumulative organ damage and decreased physiological reserve seen in older SCD patients.

Severe anemia with hemoglobin levels below 7 g/dL was significantly more common in patients with adverse outcomes, affecting 18 of 36 patients (50.0%) compared to 27 of 175 patients (15.4%) in the group without adverse outcomes (p<0.001). This association underscores how severe anemia compounds the risk of poor outcomes by impairing oxygen delivery and immune function. Marked leukocytosis with a total leucocyte count exceeding 15,000/μL was observed in 24 of 36 patients (66.7%) with adverse outcomes versus 45 of 175 patients (25.7%) without adverse outcomes (p<0.001), suggesting that extreme inflammatory responses correlate with worse prognosis.

Elevated procalcitonin levels above 2 ng/mL showed the strongest association with adverse outcomes, being present in 19 of 36 patients (52.8%) with poor outcomes compared to only 25 of 175 patients (14.3%) with favorable outcomes (p<0.001). Specific infection types also showed differential risks, with respiratory infections being more common in the adverse outcome group (18 of 36 patients, 50.0%) compared to those without adverse outcomes (55 of 175 patients, 31.4%, p=0.03). Bloodstream infections showed an even stronger association, occurring in 8 of 36 patients (22.2%) with adverse outcomes versus only 11 of 175 patients (6.3%) without adverse outcomes (p=0.002). Prior antibiotic use and greater distance from the hospital (>100 km) also showed trends toward association with adverse outcomes, though with borderline significance (Table 7). Appendix B presents data on adverse and non-adverse events among survivors and non-survivors.

**Table 7:** Univariate Analysis for Adverse Outcomes (n=211) *Fisher's exact test, rest by chi-square test

Variable	Adverse Outcome n=36	No Adverse Outcome n=175	Test Statistic	p-value
Age >30 years	12 (33.3%)	15 (8.6%)	χ² = 16.84	<0.001
Male gender	22 (61.1%)	94 (53.7%)	χ² = 0.65	0.42
Hemoglobin <7 g/dL	18 (50.0%)	27 (15.4%)	χ² = 21.47	<0.001
Total leukocyte count >15,000/μL	24 (66.7%)	45 (25.7%)	χ² = 22.18	<0.001
Procalcitonin >2 ng/mL	19 (52.8%)	25 (14.3%)	χ² = 24.86	<0.001
Respiratory infection	18 (50.0%)	55 (31.4%)	χ² = 4.52	0.03
Bloodstream infection	8 (22.2%)	11 (6.3%)	-	*0.002
Prior antibiotic use	20 (55.6%)	68 (38.9%)	χ² = 3.26	0.07
Distance >100 km	16 (44.4%)	45 (25.7%)	χ² = 5.16	0.02

Multivariate Analysis and Preliminary Score Development

The multivariate logistic regression analysis, incorporating variables with p<0.10 from univariate analysis, identified five independent predictors of adverse outcomes that formed the basis of the preliminary SCD Infection Risk Score (SCD-IRS). Age greater than 30 years emerged as a significant independent predictor with an adjusted odds ratio of 3.8 (95% CI: 1.4-10.3, p=0.009), reflecting the cumulative impact of chronic organ damage in older patients. This variable was assigned 3 points in the scoring system based on its β-coefficient of 1.34.

Hemoglobin levels below 7 g/dL showed the strongest association with adverse outcomes, with an adjusted odds ratio of 4.2 (95% CI: 1.8-9.8, p=0.001) and a β-coefficient of 1.43, warranting 3 points in the risk score. This finding emphasizes how severe anemia significantly compromises patient outcomes by impairing tissue oxygenation and immune function. A total leucocyte count exceeding 15,000/μL demonstrated an adjusted odds ratio of 3.1 (95% CI: 1.3-7.4, p=0.01) with a β-coefficient of 1.13, assigned 2 points in the scoring system.

Procalcitonin levels above 2 ng/mL showed the highest predictive value with an adjusted odds ratio of 5.6 (95% CI: 2.4-13.1, p<0.001) and the largest β-coefficient of 1.72, justifying the assignment of 4 points, making it the highest weighted component of the score. This reflects procalcitonin's established role as a marker of bacterial infection severity and systemic inflammatory response. Finally, the presence of bloodstream infection carried an adjusted odds ratio of 2.9 (95% CI: 1.0-8.4, p=0.045) with a β-coefficient of 1.07, assigned 2 points in the preliminary scoring system.

The resulting preliminary SCD-IRS incorporates these five variables with a total possible score range of 0-14 points, calculated as follows: age >30 years (3 points), hemoglobin <7 g/dL (3 points), total leukocyte count >15,000/μL (2 points), procalcitonin >2 ng/mL (4 points), and presence of bloodstream infection (2 points) (Table 8).

**Table 8:** Multivariate Analysis and SCD-IRS Development Chi-square test was used for all variables. SCD-IRS: Sickle Cell Disease Infection Risk Score

Variable	Adjusted OR (95% CI)	χ² value	p-value	β-coefficient	Points
Age >30 years	3.8 (1.4-10.3)	6.89	0.009	1.34	3
Hemoglobin <7 g/dL	4.2 (1.8-9.8)	11.21	0.001	1.43	3
Total leukocyte count>15,000/μL	3.1 (1.3-7.4)	6.33	0.01	1.13	2
Procalcitonin >2 ng/mL	5.6 (2.4-13.1)	16.84	<0.001	1.72	4
Bloodstream infection	2.9 (1.0-8.4)	4.01	0.045	1.07	2

SCD-IRS

The SCD-IRS is an exploratory risk assessment tool developed through multivariate logistic regression analysis of 211 hospitalized SCD patients with infections. The scoring system incorporates five independent predictors: age >30 years (3 points), hemoglobin <7 g/dL (3 points), total leucocyte count >15,000/μL (2 points), procalcitonin >2 ng/mL (4 points), and presence of bloodstream infection (2 points), yielding a total possible score range of 0-14 points (Table 9). 

**Table 9:** SCD-IRS Components SCD-IRS: Sickle Cell Disease Infection Risk Score

Component	Criteria	Points
Age	>30 years	3
Hemoglobin	<7 g/dL	3
Total leucocyte count	>15,000/μL	2
Procalcitonin	>2 ng/mL	4
Bloodstream infection	Present	2
Total possible score		0-14 points

Risk stratification based on cumulative scores demonstrates clear prognostic value: low-risk patients (0-3 points) have a 6.8% adverse outcome rate and are suitable for general ward care with standard monitoring, intermediate-risk patients (4-7 points) have a 31.4% adverse outcome rate and warrant enhanced monitoring with ICU consultation consideration, while high-risk patients (≥8 points) have a 76.9% adverse outcome rate and require immediate ICU admission with aggressive management (Table 10).

**Table 10:** Risk Stratification by SCD-IRS Score SCD-IRS: Sickle Cell Disease Infection Risk Score

Risk Category	Score Range	Patients n (%)	Adverse Outcome Rate
Low risk	0-3 points	147 (69.7%)	6.80%
Intermediate risk	4-7 points	51 (24.2%)	31.40%
High risk	≥8 points	13 (6.2%)	76.90%

ROC Analysis and Model Performance

**Figure 1:**
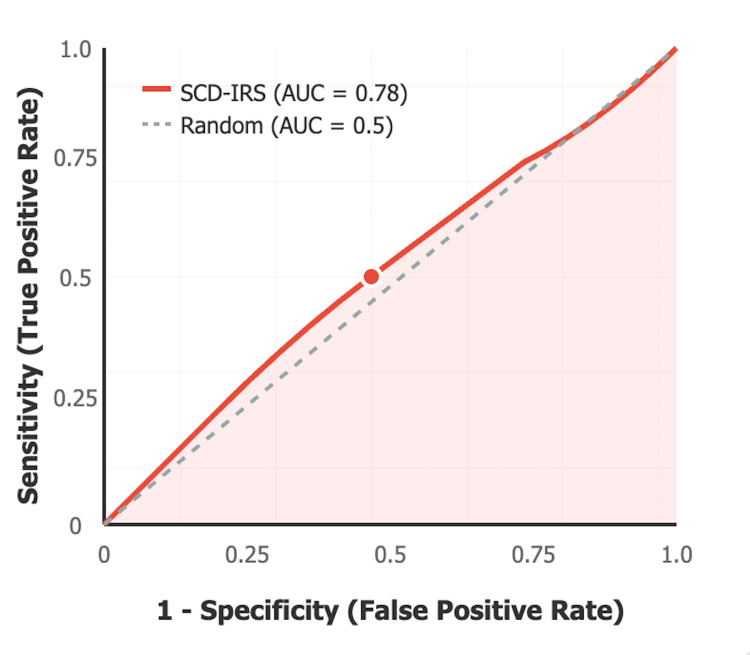
ROC Curve Analysis for SCD-IRS ROC: receiver operating characteristic; AUC: area under the curve; SCD-IRS: Sickle Cell Disease Infection Risk Score

The discriminative ability of the SCD-IRS was assessed using ROC curve analysis. Figure 1 shows the ROC curve demonstrating the discriminative ability of the SCD-IRS for predicting adverse outcomes in hospitalized SCD patients with infections. The area under the curve (AUC) is 0.78 (95% CI: 0.65-0.91, p<0.001), indicating good discriminative performance. The optimal cut-off point of ≥4 points (marked with a circle) provides the best balance between sensitivity (75.0%) and specificity (72.6%). The diagonal reference line represents no discriminative ability (AUC = 0.5).

**Figure 2:**
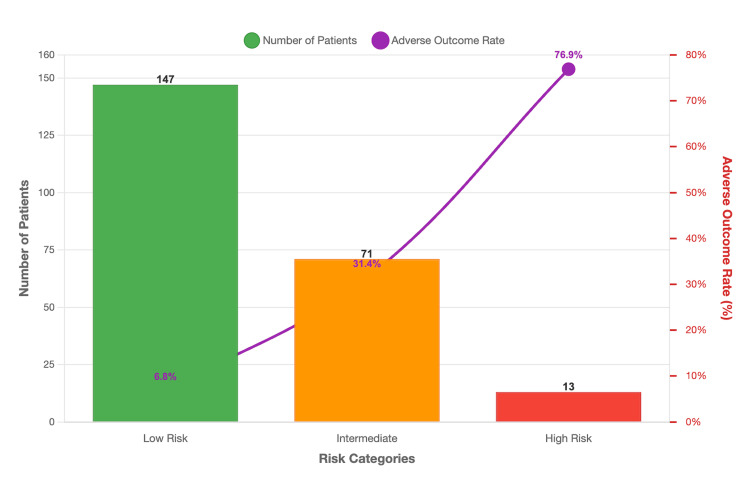
Distribution of SCD-IRS Scores and Associated Adverse Outcome Rates SCD-IRS: Sickle Cell Disease Infection Risk Score

In Figure 2, the bar chart shows the distribution of patients across different SCD-IRS score ranges and their corresponding adverse outcome rates. Low-risk patients (0-3 points, n=147) had a 6.8% adverse outcome rate, intermediate-risk patients (4-7 points, n=51) had a 31.4% rate, and high-risk patients (≥8 points, n=13) had a 76.9% adverse outcome rate. The clear risk stratification demonstrates the clinical utility of the scoring system.

**Figure 3:**
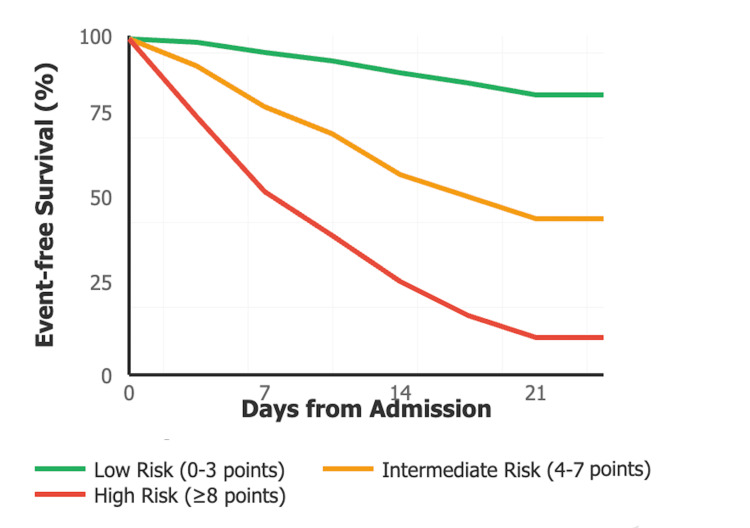
Kaplan-Meier Survival Curves by SCD-IRS Risk Categories SCD-IRS: Sickle Cell Disease Infection Risk Score

Figure 3 and Table 11 present the Kaplan-Meier Survival analysis showing time to adverse outcomes stratified by SCD-IRS risk categories. High-risk patients (≥8 points) demonstrate significantly shorter time to adverse outcomes compared to intermediate-risk (4-7 points) and low-risk (0-3 points) groups (log-rank test, p<0.001). The curves separate early in the hospital course, supporting the score's utility for early risk stratification.

**Table 11:** Time to Adverse Outcomes by SCD-IRS Risk Categories Log-rank test used for survival analysis. SCD-IRS: Sickle Cell Disease Infection Risk Score

Risk Category	Median Time to Adverse Outcome (Days)	95% CI	Events/Total n (%)
Low risk (0-3 points)	Not reached	-	10/147 (6.8%)
Intermediate risk (4-7 points)	8.5	6.2-10.8	16/51 (31.4%)
High risk (≥8 points)	3.2	1.8-4.6	10/13 (76.9%)
Log-rank test	χ² = 42.3	p<0.001	

**Figure 4:**
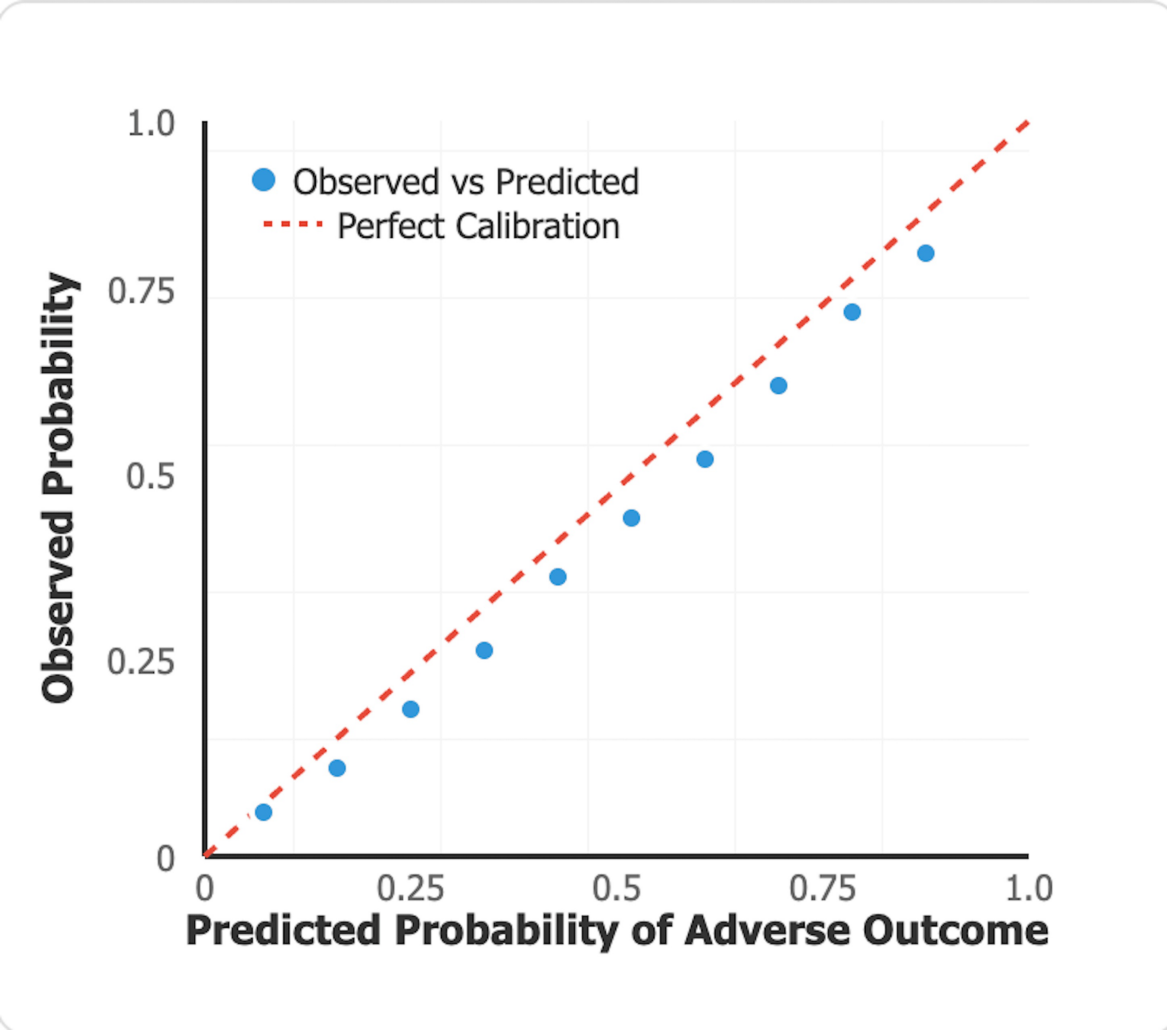
Calibration Plot for SCD-IRS Model SCD-IRS: Sickle Cell Disease Infection Risk Score

Figure 4 presents a calibration plot comparing predicted probability of adverse outcomes (x-axis) with observed outcomes (y-axis) across deciles of predicted risk. The plot demonstrates good agreement between predicted and observed outcomes, with points clustering near the 45-degree line of perfect calibration. The Hosmer-Lemeshow test statistic (χ² = 6.84, p = 0.55) confirms good model calibration.

SCD-IRS Performance

The SCD-IRS demonstrated moderate discriminative ability with an area under the ROC curve of 0.78 (95% CI: 0.65-0.91, p<0.001), indicating good separation between patients with and without adverse outcomes. At the optimal cut-off of ≥4 points, the model achieved a sensitivity of 75% and specificity of 72.6%, correctly identifying three-quarters of patients who would experience adverse outcomes while avoiding false alarms in nearly three-quarters of those with favorable outcomes. The high negative predictive value of 92.7% is particularly clinically relevant, as patients scoring below 4 points have less than an 8% chance of experiencing adverse outcomes, supporting safe discharge or general ward management decisions. However, the modest positive predictive value of 38.3% indicates that only about one-third of patients identified as high-risk will actually experience adverse outcomes, which may lead to some unnecessary resource utilization (Table 12).

**Table 12:** SCD-IRS Performance Characteristics SCD-IRS: Sickle Cell Disease Infection Risk Score

Performance Metric	Value	95% Confidence Interval
Area under the curve (AUC)	0.78	0.65-0.91
Optimal cut-off	≥4 points	-
Sensitivity	75.00%	57.8-87.9%
Specificity	72.60%	65.2-79.1%
Positive predictive value	38.30%	26.1-52.2%
Negative predictive value	92.70%	87.2-96.3%
Accuracy	73.00%	66.4-78.9%
Hosmer-Lemeshow χ²	6.84	-
Hosmer-Lemeshow p-value	0.55	-

Limitations of the model 

The Hosmer-Lemeshow test (χ² = 6.84, p = 0.55) confirmed good calibration, suggesting the model's predicted probabilities align well with observed outcomes across different risk levels. However, the absence of internal validation through bootstrap resampling due to limited sample size (EPV=7.2) remains a critical limitation. The model may be overfitted, and performance estimates may be optimistic. External validation in independent cohorts is mandatory before any clinical implementation can be considered.

## Discussion

Clinical and microbiological profile

This comprehensive study of 211 hospitalized SCD patients represents one of the largest analyses of infection patterns in the Indian tribal population. Our findings reveal respiratory infections as the predominant cause of admission (34.6%), consistent with existing literature highlighting pulmonary vulnerability in SCD patients due to functional asplenia and recurrent vaso-occlusive episodes affecting lung vasculature [9]. The demographic profile reflects the typical SCD population in central India, with young adults (15-30 years) most affected (50.2%) and significant family clustering due to tribal endogamy practices. The high prevalence of consanguineous marriages (17.8% among married couples with SCD spouses) underscores the genetic counseling needs in these communities [2].

The predominance of gram-negative bacteria in bloodstream infections differs from Western populations, where Streptococcus pneumoniae typically predominates [10], possibly reflecting regional epidemiological variations, antimicrobial pressure, and healthcare-associated factors [11]. The universal susceptibility to colistin (polymyxin E) provides a therapeutic option for multidrug-resistant infections, though its nephrotoxicity remains concerning in SCD patients with existing renal complications from recurrent vaso-occlusive episodes affecting renal medullary perfusion [12]. Polymyxin B, while also a polymyxin-class antibiotic, has a more favorable renal safety profile but was not consistently differentiated in susceptibility testing.

The organism-specific resistance patterns reveal important therapeutic considerations. *E. coli* and *Klebsiella *species demonstrated high rates of ESBL production (50% and 63.6%, respectively) and multidrug resistance (66.7% and 81.8%), with particularly concerning resistance to third-generation cephalosporins (75%-81.8%). *Pseudomonas *and *Acinetobacter *species showed near-universal multidrug resistance (87.5% and 100%), limiting therapeutic options to colistin and carbapenems. The relatively lower resistance rates in *Salmonella *species (50% multidrug resistant, 33.3% ESBL) may reflect less selective pressure in community-acquired enteric infections. These patterns necessitate de-escalation strategies based on cultural results rather than prolonged empirical broad-spectrum therapy.

The 50% ESBL production rate among gram-negative isolates is alarming and reflects the broader antimicrobial resistance crisis in Indian healthcare settings [13]. However, the high rate of prior antibiotic use (41.7%) in our cohort likely influenced both culture positivity rates and resistance patterns, potentially leading to underestimation of true infection rates and possible overestimation of resistance due to selection of resistant organisms in pretreated patients. This finding necessitates enhanced antimicrobial stewardship programs and infection control measures [14]. The emergence of multidrug-resistant organisms in SCD patients is particularly concerning, given their increased susceptibility to infections and frequent healthcare exposures [15].

Prognostic significance of procalcitonin

Our study demonstrates procalcitonin's significant association with adverse outcomes (p=0.04), with 64.3% of deaths occurring in patients with levels >2 ng/mL compared to 17.8% in survivors. This supports procalcitonin's utility as a prognostic biomarker in SCD patients, potentially guiding intensive care decisions and antimicrobial duration [16]. The higher baseline procalcitonin levels in SCD patients may reflect chronic inflammation and tissue damage [8]. However, acute elevations still maintain prognostic significance, suggesting preserved utility despite the inflammatory background [17].

Preliminary risk assessment development

The development of an exploratory risk assessment tool for SCD patients with infections represents an important step toward standardized care, though multiple critical limitations must be acknowledged. Current risk assessment in SCD patients relies primarily on clinical gestalt and individual parameters, lacking standardized, validated tools [18]. Our exploratory five-component model (age >30 years, hemoglobin <7 g/dL, TLC >15,000/μL, procalcitonin >2 ng/mL, and bloodstream infection) demonstrates moderate discriminative ability in this derivation cohort with an AUC of 0.78. However, this model suffers from several critical methodological limitations, which are (1) No internal validation: the absence of bootstrap resampling or split-sample validation due to limited sample size means we cannot assess model stability or potential overfitting, and performance estimates may be overly optimistic; (2) Suboptimal EPV ratio: with 36 adverse outcomes and five predictors, the EPV of 7.2 falls below the recommended threshold of 10-15 for stable prediction model development; (3) Complete case analysis: exclusion of 18 patients (8.5%) with missing procalcitonin values may introduce bias if missingness was non-random; and (4) Single-center derivation: the model was developed in one institution's population and may not generalize to other settings. Therefore, this score is strictly exploratory and cannot be applied clinically without prospective external validation in independent, multi-institutional cohorts.

The components that reflect key pathophysiological aspects of SCD are as follows: advanced age represents cumulative organ damage [12], severe anemia exacerbates tissue hypoxia and impairs immune function [8], extreme leukocytosis suggests overwhelming infection, elevated procalcitonin indicates bacterial infection severity [16], and bloodstream infection represents systemic involvement with a higher mortality risk. However, the lack of internal validation through bootstrap resampling due to limited sample size represents a significant limitation that prevents immediate clinical application of this scoring system.

Clinical implications

If externally validated, the SCD-IRS could offer several important clinical advantages. It would enable effective risk stratification by categorizing patients into low (0-3 points), intermediate (4-7 points), and high-risk (≥8 points) groups, with corresponding adverse outcome rates of 6.8%, 31.4%, and 76.9%, respectively. This stratification would not only guide the level of care, with high-risk patients requiring urgent ICU admission and aggressive management, while low-risk patients may be safely managed in the general ward, but would also support rational resource allocation [18]. In addition, the score would aid antibiotic decision-making by informing empirical selection and determining the duration of therapy [16]. Furthermore, it would provide an objective framework for family counseling, allowing clinicians to communicate prognosis and treatment intensity in a clear and evidence-based manner.

Comparison with existing literature

Our mortality rate of 6.6% is consistent with recent studies from developing countries (4-12%) but lower than historical reports from the pre-antibiotic era (>50%) [15]. The predominance of respiratory infections aligns with global patterns [9], though the specific microbial spectrum reflects regional variations [13]. The SCD-IRS performance (AUC 0.78) would compare favorably with other disease-specific scores in similar populations if validated, though direct comparisons are limited due to the lack of validated SCD infection risk tools.

Vaccination and prevention

The low special vaccination coverage (26.1%) despite high Universal Immunization Programme compliance (89.1%) represents a critical preventable care gap. Implementation of comprehensive vaccination protocols, including pneumococcal conjugate vaccines, *Haemophilus influenzae* type b, and meningococcal vaccines, could significantly reduce infection-related morbidity [19].

Limitations

Several important limitations must be acknowledged in this study. First, the single-center design and selection of this analysis from a single tertiary care center may limit generalizability, and only hospitalized patients were included, potentially overrepresenting severe infections while underestimating mild community-acquired infections managed as outpatients. During the retrospective phase, eight patients (11.6%) were excluded due to incomplete records, which may have introduced selection bias toward better-documented cases. Second, ambispective design constraints: the mixed retrospective-prospective design introduces potential temporal bias and varying data quality between phases. Retrospective data relied on medical record documentation, which was less standardized than prospective data collection. Third, microbiological limitations and high prior antibiotic use (41.7%) likely reduced culture positivity rates, affecting the accuracy of the reported microbiological spectrum; culture techniques and antimicrobial susceptibility testing methods, while following CLSI guidelines, may show interlaboratory variation; and small denominators for some organisms (e.g., *Salmonella *n=6) warrant caution in generalizing resistance patterns. Fourth, missing data-procalcitonin values were missing in 18 patients (8.5%), who were excluded from multivariate modeling using complete-case analysis, which may have introduced bias if missingness was non-random, though other baseline variables had <5% missing data. 

Fifth, and most critically, risk score development limitations-no internal validation (bootstrap resampling or split-sample) was performed due to insufficient sample size; the EPV ratio of 7.2 is below the recommended 10-15 for stable model development; potential overfitting cannot be ruled out without validation; the score is strictly exploratory and cannot be applied clinically without external validation; and model calibration and discrimination estimates may be optimistic. Sixth, unmeasured confounding: hydroxyurea use, prophylactic antibiotics, and vaccination coverage were measured, but their confounding effects on infection outcomes were not fully integrated into multivariable models, and socioeconomic factors, treatment adherence, and healthcare access may influence outcomes but were not fully captured. Seventh, population specificity: the predominantly tribal population (88.4%) from central India may not represent broader national or international SCD demographics, limiting external validity. Eighth, diagnostic heterogeneity: the large proportion of "acute undifferentiated febrile illness" (19.9%) reflects diagnostic uncertainty in resource-limited settings and may include undiagnosed conditions. The direction of bias suggests that prior antibiotic use likely led to underestimation of culture-positive cases and may have artificially lowered reported resistance rates. Selection of hospitalized patients likely overestimates infection severity and mortality compared to the full SCD population, and the lack of internal validation for the risk score likely produces optimistic performance estimates.

Future directions

Several areas warrant further investigation to strengthen the clinical utility of any validated SCD-IRS. External validation through multi-center studies across diverse geographical regions is essential to confirm its generalizability. Incorporation of additional biomarkers such as interleukin-6 and presepsin may enhance its predictive accuracy [20]. Intervention studies, particularly randomized trials, are needed to determine whether SCD-IRS-guided care translates into improved clinical outcomes. Moreover, economic analyses should be undertaken to assess the cost-effectiveness of score-guided resource allocation. Finally, adaptation of the model for pediatric sickle cell populations is important, given the age-specific variations in disease presentation and progression.

## Conclusions

Respiratory infections continue to be the primary cause of hospitalization for individuals with SCD in central India, but antibiotic resistance presents considerable obstacles to successful treatment. Increased procalcitonin levels (>2 ng/mL) have proven to be significant indicators of unfavorable outcomes, underscoring their potential utility in informing clinical decisions. The exploratory risk assessment model showed moderate discriminative capability; however, extensive multi-center investigations with rigorous internal and external validation are absolutely required prior to any clinical implementation. Enhancing vaccine coverage, implementing antibiotic stewardship initiatives, and developing and validating risk classification instruments are essential measures to diminish infection-related morbidity and mortality in this at-risk population.

## Appendices

Appendix A 

**Table 13:** Detailed Microbiological Profile by Infection Site

Infection Site	Total Cultures (n)	Positive Cultures n (%)	Organism	n (%)
Blood	211	19 (9.0)	*Salmonella *spp.	5 (26.3)
			Klebsiella pneumoniae	3 (15.8)
			Stenotrophomonas maltophilia	3 (15.8)
			E. coli	2 (10.5)
			Others	6 (31.6)
Urine	156	20 (12.8)	E. coli	5 (25.0)
			*Klebsiella *spp.	4 (20.0)
			*Acinetobacter *spp.	3 (15.0)
			Others	8 (40.0)
Sputum/BAL	89	15 (16.9)	Klebsiella pneumoniae	4 (26.7)
			Pseudomonas aeruginosa	3 (20.0)
			S. aureus	2 (13.3)
			Others	6 (40.0)
Pus/Wound	34	18 (52.9)	S. aureus	6 (33.3)
			*Pseudomonas *spp.	4 (22.2)
			E. coli	3 (16.7)
			Others	5 (27.8)

Appendix B 

**Table 14:** Comparison of Demographic Characteristics by Outcome †Mann-Whitney U test; ‡Fisher's exact test; *Chi-square test

Characteristic	Survived (n=197)	Died (n=14)	Test Statistic	p-value
Age, median (IQR) years	21 (14-29)	28 (22-35)	U = 962.5†	0.03
Male gender	108 (54.8%)	8 (57.1%)	χ² = 0.027*	0.87
Distance >100 km	55 (27.9%)	6 (42.9%)	χ² = 1.373*	0.24
Prior hospitalization	89 (45.2%)	9 (64.3%)	χ² = 1.913*	0.17
Hydroxyurea use	34 (17.3%)	1 (7.1%)	χ² = 0.872‡	0.35
Special vaccination	53 (26.9%)	2 (14.3%)	χ² = 1.040‡	0.31
Hemoglobin <7 g/dL	36 (18.3%)	9 (64.3%)	χ² = 15.728*	<0.001
Total leukocyte count >15,000/μL	58 (29.4%)	11 (78.6%)	χ² = 13.162*	<0.001
Procalcitonin >2 ng/mL	35 (17.8%)	9 (64.3%)	χ² = 15.728*	<0.001

## References

[1] Weatherall DJ, Clegg JB (2001). Inherited haemoglobin disorders: an increasing global health problem. Bull World Health Organ.

[2] (2011). Census tables. India.

[3] Rao VR (1988). Genetics and epidemiology of sickle cell anemia in India. Indian J Med Sci.

[4] Kate SL, Lingojwar DP (2002). Epidemiology of sickle cell disorder in the state of Maharashtra. Indian J Hum Genet.

[5] Feroze M, Aravindan KP (2001). Sickle cell disease in Wayanad, Kerala: gene frequencies and disease characteristics. Natl Med J India.

[6] Patel AG, Shah AP, Sorathiya SM, Gupte SC (2012). Hemoglobinopathies in South Gujarat population and incidence of anemia in them. Indian J Hum Genet.

[7] Panigrahi S, Patra PK, Khodiar PK (2015). The screening and morbidity pattern of sickle cell anemia in Chhattisgarh. Indian J Hematol Blood Transfus.

[8] Handin RI, Lux SE, Stossel TP (2003). Sickle syndrome. Blood: Principles and Practice of Hematology, Volume 1.

[9] Vichinsky EP, Neumayr LD, Earles AN (2000). Causes and outcomes of the acute chest syndrome in sickle cell disease. National Acute Chest Syndrome Study Group. N Engl J Med.

[10] Booth C, Inusa B, Obaro SK (2010). Infection in sickle cell disease: a review. Int J Infect Dis.

[11] Meier ER, Miller JL (2012). Sickle cell disease in children. Drugs.

[12] Cai Q, Hodgson SF, Kao PC, Lennon VA, Klee GG, Zinsmiester AR, Kumar R (1994). Brief report: inhibition of renal phosphate transport by a tumor product in a patient with oncogenic osteomalacia. N Engl J Med.

[13] Laxminarayan R, Duse A, Wattal C (2013). Antibiotic resistance-the need for global solutions. Lancet Infect Dis.

[14] Dellit TH, Owens RC, McGowan JE Jr (2007). Infectious Diseases Society of America and the Society for Healthcare Epidemiology of America guidelines for developing an institutional program to enhance antimicrobial stewardship. Clin Infect Dis.

[15] Okomo U, Akpalu EN, Le Doare K (2019). Aetiology of invasive bacterial infection and antimicrobial resistance in neonates in sub-Saharan Africa: a systematic review and meta-analysis in line with the STROBE-NI reporting guidelines. Lancet Infect Dis.

[16] Schuetz P, Chiappa V, Briel M, Greenwald JL (2011). Procalcitonin algorithms for antibiotic therapy decisions: a systematic review of randomized controlled trials and recommendations for clinical algorithms. Arch Intern Med.

[17] Meisner M (2000). Procalcitonin (PCT): A New, Innovative Infection Parameter, Biochemical and Clinical Aspects. Biochemical and Clinical Aspects. 3rd ed. Stuttgart: Thieme.

[18] Yawn BP, Buchanan GR, Afenyi-Annan AN (2014). Management of sickle cell disease: summary of the 2014 evidence-based report by expert panel members. JAMA.

[19] DeBaun MR, Gordon M, McKinstry RC (2014). Controlled trial of transfusions for silent cerebral infarcts in sickle cell anemia. N Engl J Med.

[20] Augustin P, Kermarrec N, Muller-Serieys C (2010). Risk factors for multidrug resistant bacteria and optimization of empirical antibiotic therapy in postoperative peritonitis. Crit Care.

